# The Effects of Smoking, Alcohol, and Dietary Habits on the Progression and Management of Spondyloarthritis

**DOI:** 10.3390/jpm14121114

**Published:** 2024-11-21

**Authors:** Mauro Fatica, Eneida Çela, Mario Ferraioli, Luisa Costa, Paola Conigliaro, Alberto Bergamini, Francesco Caso, Maria Sole Chimenti

**Affiliations:** 1 Rheumatology, Allergology and Clinical Immunology, Department of Systems Medicine, University of Rom Tor Vergata, 00133 Rome, Italy; maufat25@gmail.com (M.F.); eneida.cela@students.uniroma2.eu (E.Ç.); mario.ferraioli@students.uniroma2.eu (M.F.); paola.conigliaro@uniroma2.it (P.C.); bergamini@med.uniroma2.it (A.B.); 2 Rheumatology Research Unit, Department of Clinical Medicine and Surgery, School of Medicine and Surgery, University of Naples Federico II, 80131 Naples, Italy; lv.costa@libero.it (L.C.); francescocaso1@yahoo.it (F.C.)

**Keywords:** Spondyloarthritis, smoking, alcohol, dietary habits

## Abstract

Spondyloarthritis (SpA) is a group of chronic inflammatory diseases affecting the spine and peripheral joints, causing pain, stiffness, and reduced mobility. This narrative review examines how lifestyle factors—specifically smoking, alcohol consumption, and unhealthy diet—contribute to the onset and progression of SpA. It highlights their impact on disease activity, comorbidities, radiographic damage, and treatment response. Therefore, healthcare providers are encouraged to support patients in making personalized lifestyle changes. These findings underscore the importance of a comprehensive approach to SpA management, integrating lifestyle modifications with conventional therapies for optimal disease control and improved outcomes.

## 1. Introduction

Spondyloarthritis (SpA) is a heterogeneous group of chronic inflammatory arthritis that can primarily involve the spine and sacroiliac joints (axial-SpA), as well as the limbs and other peripheral joints outside the spine (peripheral-SpA) [1]. The pathogenesis of SpA encompasses a complex interplay of genetic, environmental, and immunological factors, which trigger the action of pro-inflammatory cytokines such as Tumor Necrosis Factor Alpha (TNF-α), interleukin (IL)-23, and IL-17. These cytokines are crucial for the onset of the disease and the development of its various clinical manifestations [1,2,3].

Impaired bone metabolism involving both osteoclasts and osteoblasts has been identified in patients with SpA [2]. This imbalance can result in the development of bone lesions, occurring both peripherally and, more prominently, axially. Magnetic Resonance Imaging (MRI) studies have provided a detailed characterization of these lesions, which are typically classified into active (or inflammatory) lesions, indicated by bone marrow edema at various sites, and structural (or post-inflammatory) lesions, primarily represented by bone erosions, focal fat deposition, bone spurs, and ankylosis [4]. These structural changes can significantly reduce spinal flexibility, impair mobility, and ultimately diminish the quality of life for affected individuals. Moreover, SpA can be associated with psoriasis (PsO), inflammatory bowel diseases (IBD), non-infectious uveitis (NIU), and a range of comorbidities, including metabolic syndrome (MetS), non-alcoholic fatty liver disease (NAFLD), and mental health disorders [5,6,7].

The diagnosis of SpA is performed through anamnestic and clinical evaluations, along with laboratory findings [mainly high C-reactive protein (CRP) levels] and imaging techniques, such as ultrasound, X-ray, and MRI [8]. A short-term course of non-steroidal anti-inflammatory drugs (NSAIDs) or intra-articular corticosteroids are used to alleviate symptoms during disease flares [9,10]. Beyond this effect, recent studies suggest that regular NSAID use may help prevent syndesmophyte formation by controlling inflammation and lowering CRP levels, a recognized biomarker of structural damage and disease progression [11]. Conventional disease-modifying antirheumatic drugs (csDMARDs) such as methotrexate (MTX) or sulphasalazine (SSZ) are used exclusively for the treatment of joint peripheral manifestations in SpA patients [9,10]. For axial forms or in cases of peripheral involvement refractory to csDMARD therapy, biologic drugs (TNF inhibitors, IL-12/23 inhibitors, IL-23 inhibitors, IL-17A inhibitors, and IL-17A/F inhibitors) and small molecules (Janus Kinase inhibitors and phosphodiesterase 4 inhibitors) are usually administered [9,10,11,12,13].

These drugs offer the potential to control the activation of molecular pathways crucial for the development of SpA, allowing for the reduction of inflammatory state, mitigation of joint damage, management of comorbidities, and improvement in quality of life. However, not all patients are able to achieve remission or low disease activity with these pharmacological treatments. This could result from the heterogeneity of SpA in terms of phenotypic presentation, severity, presence of comorbidities, diagnostic delay, and poor adherence to therapy due to side effects or psychosocial factors. Moreover, it has been observed how the combination of multiple unhealthy lifestyle habits (e.g., cigarette smoking, obesity, lack of physical activity, etc.) can result not only in poor control of joint disease activity but also significantly impact quality of life and promote the development of impaired physical and mental health in SpA patients [14,15].

It is therefore of fundamental importance that patients discuss with their rheumatologists the possibility of modifying unhealthy aspects of their lifestyle, in accordance with the principles of international recommendations [16,17]. In this context, this narrative review examines the impact of unhealthy habits such as smoking, alcohol, and an unbalanced diet on the onset and progression of SpA, particularly psoriatic arthritis (PsA) and axial-SpA (AxSpA), and how a holistic approach that manages lifestyle factors, complementing pharmacological therapy, can theoretically offer greater possibilities to control the disease.

## 2. Smoking

The causal link between smoking and the risk of developing SpA has been supported by less robust evidence compared to other immune-mediated rheumatic diseases, such as rheumatoid arthritis (RA) or systemic lupus erythematosus (SLE). In these diseases, the correlation is primarily attributed to smoking’s role in promoting the activation of autoreactive B cells, which produce a range of autoantibodies crucial for both disease onset and the development of a typically more aggressive phenotype [18]. The lack of a significant impact from autoantibodies in the pathophysiology of SpA might suggest a weaker theoretical association between smoking and SpA onset.

However, smoking may serve as an environmental trigger that interacts with genetic factors to both initiate and exacerbate SpA (data are summarized in Table 1). While the exact mechanisms are complex, smoking generally induces immune dysregulation and inflammation in genetically predisposed individuals. Specifically, smoking introduces harmful substances such as nicotine, carbon monoxide, and free radicals into the body, which stimulate the release of cytokines that induce inflammation and the development of structural damage [19]. The free radicals from cigarette smoke cause oxidative stress, which damages cells and tissues. This stress can lead to the activation of the nuclear factor kappa-light-chain-enhancer of activated B cells (NF-kB), a transcription factor that further increases the production of pro-inflammatory cytokines, through the modulation of transcription of a series of genes involved in the inflammatory response [20].

Moreover, smoking disrupts normal immune system function by affecting T cells, macrophages, and dendritic cells. In this regard, it can increase the proportion of Th17 cells while impairing function and reducing the number of regulatory T cells (Tregs), which are crucial for maintaining immune tolerance and preventing autoimmune responses [21]. Smoking can also impair joint health through indirect effects. In fact, it negatively impacts bone metabolism, leading to reduced bone mineral density and increased risk of osteoporosis, enhancing disease progression and structural damage [22]. Finally, smoking can affect vascular function as well, leading to endothelial dysfunction and poor blood flow to the joints and entheses, worsening inflammation and hindering the repair processes in these areas [23].

Given these numerous mechanisms potentially promoting the development of SpA, evidence of a higher incidence of these diseases among smokers comes from observational studies. In this context, a Norwegian case-control study found that self-reported cases of incident AxSpA were significantly higher among current or former smokers compared to never-smokers [24]. Nevertheless, no correlation was found between AxSpA incidence and the number of pack years. Thus, the authors emphasized that the association between smoking and AxSpA development did not establish a definitive causal relationship. A large UK study demonstrated that current or former smoking habits were associated with approximately a 30% increased risk of developing PsA compared to the general population, proportional to the number of pack years [25].

However, the relationship between smoking and the development of PsO and PsA has been debated over time, leading to the conceptualization of the “smoking paradox”, which refers to the counterintuitive relationship between smoking and the incidence and severity of these conditions. Specifically, smoking has been shown to increase the risk of developing PsO in a dose-dependent manner, with greater numbers of cigarettes smoked daily correlating with higher risk. Smoking also exacerbates the severity of PsO and can negatively impact treatment responses, often making the condition more challenging to manage [26,27].

On the other hand, in some studies smoking appeared to be paradoxically associated with a lower incidence of developing PsA among individuals with PsO: in particular, in a metanalysis by Gazel and colleagues, smoking habit had an odds ratio (OR) of 0.7 in PsO patients and an OR 1.1 in the general population [49]. The key to the correct interpretation of these conflicting observations, despite various biological and molecular hypotheses proposed, appears to be primarily methodological [50]. The effect of smoking on the risk of PsA seems to be mediated almost entirely through the effect of smoking on PsO. In fact, by analyzing only PsO patients, Nguyen and colleagues showed that the hazard ratio for PsA in smokers versus non-smokers drops from 1.27 in the general population to 0.91 in individuals with PsO [25]. The smoking paradox in PsO and PsA highlights the complex and sometimes contradictory effects of smoking on autoimmune and inflammatory conditions. While smoking generally worsens PsO, it appears to reduce the risk of developing PsA among those with PsO. However, understanding this phenomenon requires further research into the underlying biological mechanisms and the interplay between environmental and genetic factors.

In addition to PsO, smoking has been correlated with a higher prevalence of other extra-articular manifestations of SpA and comorbidities, particularly cardiometabolic disorders and osteoporosis [28]. Smoking significantly impacts disease burden as well. Data from the DESIR cohort show that smokers with early AxSpA developed symptoms, particularly inflammatory low back pain, about 1.5 years earlier than non-smokers. Smokers also exhibited greater disease activity and a greater impact on quality of life [29]. Indeed, smokers with SpA frequently experience higher levels of disease activity and more severe symptoms, including increased pain, stiffness, and functional impairment. The inflammatory effects of smoking can promote the progression of structural damage, especially in the spine [28,30]. In fact, research indicates a dose-dependent relationship between smoking and increased disease severity, which is reflected in greater radiographic progression. A study involving 210 AxSpA patients found a significant increase in the modified Stoke Ankylosing Spondylitis Spine Score (mSASSS) of ≥2 units over two years in 28.6% of smokers consuming more than 10 cigarettes per day, compared to just 10.1% of non-smokers. Additionally, smokers with higher cigarette consumption exhibited increased levels of CRP, indicating heightened inflammatory activity, which is believed to contribute to the radiographic progression [31]. Furthermore, a comprehensive analysis revealed that smoking not only exacerbates functional impairment and inflammatory markers but also results in more severe radiographic outcomes in AxSpA patients. In fact, smokers had a higher incidence of spinal fusion and other radiographic changes compared to non-smokers, as well as higher disease activity [Ankylosing Spondylitis Disease Activity Score (ASDAS), Bath Ankylosing Spondylitis Disease Activity Index (BASDAI)], higher Bath Ankylosing Spondylitis Functional Index (BASFI) and Bath Ankylosing Spondylitis Metrology Index (BASMI) [32].

On the other hand, a Spanish post hoc comparative analysis of multicenter observational studies showed no significant association between smoking and mSASSS score [33]. The authors suggest that occupational characteristics of SpA patients might be a more significant factor in radiographic progression than smoking itself. In fact, smoking is more prevalent in blue-collar jobs, which often involve physically demanding work, as well as in various lifestyle and socioeconomic conditions, including unemployment, lower physical activity, lower educational levels, and higher BMI [51]. Indeed, these factors may contribute to the progression of radiographic damage, independent of smoking habits [51].

While strong and consistent evidence highlights the negative impact of smoking on axial involvement in SpA, this effect does not necessarily extend to peripheral involvement. The multinational cross-sectional ASAS-PerSpA study, which included 4181 patients with AxSpA, peripheral SpA, or PsA, found that smoking in AxSpA patients was associated with a lower prevalence of peripheral arthritis and enthesitis [37].

Finally, smoking can impair the response to treatment, especially to biologic drugs. A systematic review found inconsistent evidence regarding the impact of smoking on TNF inhibitor efficacy in patients with AxSpA [34]. Of the six studies included, only two found a negative influence of smoking in response to treatment, particularly highlighting substantially lower odds of achieving BASDAI 50 response after 12 months of treatment for smokers and ex-smokers compared to non-smoker AxSpA patients [35,36]. This effect may derive not only from the capacity of smoke to trigger inflammation and consequently worsen the disease activity but also from the possible promotion of the formation of neutralizing anti-TNF-drug antibodies [52]. Regarding the IL-17A inhibitor secukinumab, one study found that the reduction in the ASDAS-CRP score from baseline to week 6 was negatively correlated with smoking habits [53].

For the other drugs currently available for SpA treatment, data primarily come from studies on PsO patients. Smokers treated with ixekizumab and risankizumab had a lower likelihood of achieving a Dermatology Life Quality Index (DLQI) score of 0/1 and a Psoriasis Area Severity Index (PASI) 100 response, respectively [54,55]. Conversely, current evidence does not strongly support the significant impact of smoking on the efficacy of ustekinumab and guselkumab [56,57].

## 3. Alcohol

Overall, research on alcohol’s effects on inflammation frequently yields surprising and inconsistent findings (data are summarized in Table 1). In fact, it has been noted that modest amounts of ethanol can suppress the production of pro-inflammatory cytokines such as TNF and IL-6 in vitro, as well as lower levels of these molecules and CRP were detected in mild-drinker adults [38]. Ethanol can also have immunosuppressive effects, through various mechanisms that mainly include the impaired function of immune cells and the weakening of the cutaneous–mucosal epithelial barriers [39].

Therefore, it has long been supported that alcohol may have potential protective roles in the development of immune-mediated and autoimmune diseases, including inflammatory arthritis. However, population studies generally reveal a U-shaped relationship between alcohol intake and inflammation. While moderate drinking appears to reduce inflammation, this protective effect disappears as alcohol consumption increases [40]. In fact, chronic alcohol consumption can increase the risk of developing PsO in the general population and worsen the condition in those already affected. Studies using murine models have shown that alcohol promotes keratinocyte proliferation, induces T-helper cells (Th1/Th17) to release pro-inflammatory cytokines, and activates the JAK-STAT pathway [45].

Moreover, although the data derive from a study in which PsA diagnosis was self-reported, the development of PsA was observed to be significantly associated with alcohol consumption in a large cohort of 82,672 US women. Specifically, the authors observed a 43% increased risk of PsA with a cumulative average alcohol intake of more than 15 g/day, and an approximately fourfold increase in risk when the intake was at least 30 g/day [46]. Chronic alcohol intake can significantly impair the quality of life for SpA patients as well. Alcohol negatively impacts bone health by disrupting the balance between bone formation and resorption, leading to compromised bone density and an increased risk of fractures. Additionally, it can interfere with the absorption of micronutrients and may interact with medications such as NSAIDs or DMARDs, potentially diminishing their effectiveness or increasing the risk of adverse effects, including gastrointestinal bleeding and liver damage [47]. In a study conducted on 278 AxSpA patients, alcohol consumption, independently from quantity, has demonstrated to be a predictive factor for the progression of spinal structural damage, being correlated with new syndesmophytes formation, progression of pre-existing syndesmophytes, and with mSASSS changes ≥2 units [48].

Regarding the impact on SpA disease activity, current evidence is still controversial. A study involving 979 patients with various types of inflammatory arthritis, including SpA, found that both male gender and risky drinking (over 15 units of alcohol per week) were significantly associated with remission [58]. However, after adjusting for gender, alcohol consumption was not significantly linked to disease activity. In contrast, when adjusting for alcohol consumption, gender remained a significant factor influencing disease activity. This suggests that, although men with inflammatory arthritis consume generally more alcohol than women, their overall disease activity is less severe, likely due to gender rather than drinking habits. Indeed, the analysis on the large ASAS-PerSpA Cohort has demonstrated how alcohol consumption was associated with a lower prevalence of currently active peripheral arthritis and enthesitis [37]. Moreover, a Chinese cross-sectional study evidenced that, while smoking effect on AxSpA patients was always detrimental, alcohol had a complex relationship with disease activity [41]. In fact, compared to non-drinkers, while moderate alcohol intake (≤25 g/day) did not significantly increase inflammatory markers, the intensity of morning stiffness and BASDAI score, heavy drinking (>25 g/day) was associated with worse outcomes (especially BASMI), similar to the effects of smoking. In addition, patients who both smoked and consumed alcohol showed the highest levels of disease activity and the worst physical functioning, suggesting a cumulative synergistic negative effect on AxSpA [41].

Another cross-sectional study on 229 AxSpA patients found a correlation between alcohol consumption and lower disease activity [42]. Moderate alcohol drinking patients (≤25 g/day, 64% of the cohort) had reduced inflammation and better disease outcomes including ASDAS, BASDAI, and BASFI scores compared with non-drinkers. However, the authors caution against overgeneralizing these findings, as several biases may affect the accurate interpretation of results. These include the elevated pain threshold induced by alcohol consumption and the tendency to focus solely on the amount of alcohol consumed, without considering factors such as the frequency of intake (e.g., occasional, constant, or binge drinking) or the specific type of alcoholic beverage [43]. In fact, the protective effects of moderate consumption of certain beverages, such as red wine, may not be primarily due to ethanol, but rather to other beneficial compounds it contains, such as resveratrol, flavonoids, and polyphenols. Notably, these substances can induce significant cardioprotective effects and reduce systemic inflammation, by inhibiting the action of proinflammatory cytokines, through attenuation of their intracellular signaling pathways [44].

## 4. Dietary Habits

### 4.1. Impact of Diet and Excessive Weight on SpA Pathogenesis and Outcomes

This section focuses on individuals without co-existing IBD. In fact, while there is a recognized interplay between diet, gut microbiota, and the immune system in SpA, the presence of IBD introduces confounding additional variables. Specifically, IBD itself significantly impacts both microbiota composition and dietary habits, making it challenging to isolate the effects and repercussions attributable solely to SpA [59]. Unbalanced diets (e.g., high in calories, saturated fats, and refined sugars, while being low in fiber, whole grains, and plant-based foods), both in the general population or among SpA patients, are broadly linked to an increased risk of developing conditions such as diabetes, cardiovascular diseases, and even cancer [60]. Additionally, unbalanced diets can lead to deficiencies in micronutrients like vitamin D, folic acid, and vitamin B12, potentially exacerbating the severity of the disease [61].

Most of the works in the literature focus on the role of overweight and obesity in promoting SpA development of various forms of SpA, particularly PsA. This association is further supported by the higher prevalence of these conditions in patients at risk of developing SpA, such as those with PsO, compared to the general population [62]. In this regard, there is strong evidence supporting obesity as a risk factor for the development of PsA. Indeed, the European Alliance of Associations for Rheumatology (EULAR) has recently included obesity as a high-risk condition for promoting the transition from PsO to PsA [63]. This risk primarily arises from an excess of visceral adipose tissue, which not only increases the biomechanical load on spine, joints, and tendinous–ligamentous structures of the lower limbs but, more critically, plays a significant role through its cellular secretory function [64,65]. In particular, it has been documented that obese patients have increased serum levels of resistin, leptin, and several pro-inflammatory cytokines (mainly TNF, IL-23, and IL-17), which are crucial for the pathogenesis of SpA [66].

Obesity promotes the development of a typically more aggressive disease phenotype, with higher disease activity, increased serum levels of CRP, greater impact on quality of life, and reduced response to treatment, particularly to TNF inhibitors [6]. This correlation can also be observed inversely, as weight loss can theoretically reduce the onset of PsA in at-risk individuals based on these humoral and biomechanical mechanisms, as well as mitigate joint inflammation, offering greater opportunities for disease control [67,68,69].

Additionally, unhealthy dietary habits can contribute to the development or exacerbation of SpA, independently from excessive adiposity. For instance, it is well established that mouse models fed with a Western diet (high in saturated fats and refined sugar) displayed increased inflammation in the skin and joints. This inflammation is driven by elevated levels of IL-23, which are promoted by significant detrimental changes in the gut microbiota composition [70].

Based on this evidence, various dietary interventions could be beneficial not only for obese patients seeking to lose weight and manage disease activity, but also for individuals of normal weight (data are summarized in Table 2) [71,72,73].

### 4.2. Mediterranean Diet

The Mediterranean diet (MD) is a dietary pattern inspired by the traditional eating habits of people living in the Mediterranean region, particularly Southern Italy, Greece, and Spain. Its core principles include plant-based food, healthy fats (particularly omega-3), moderate lean protein intake, little consumption of red meat, sweets, and alcohol [74]. This dietary regimen is known for its numerous health benefits, including weight management, diabetes. Similarly, MD promotes prevention of some chronic diseases such as cardiovascular and cognitive ones, as well as longevity [75].

An Italian monocentric study involving 161 patients with AxSpA found that those following MD showed significant improvements in disease activity [76]. Specifically, adherence to the diet was associated with a ≥20% reduction in the ASDAS-CRP after 6 months. Similarly, an Italian multicentric study on 211 PsA patients documented that although CRP levels were independent of the degree of adherence to MD, measured with the PREDIMED questionnaire, a correlation between patients who had poor adherence and high Disease Activity index in PSoriatic Arthritis (DAPSA) score [77]. In a recent cross-sectional study involving 279 PsA patients and 76 PsO patients, Katsimbri and colleagues investigated the impact of MD and exercise on disease control [78]. The authors highlighted that higher adherence to the Mediterranean diet was primarily associated with a reduction in skin disease activity, particularly with significantly reduced PASI and Body Surface Area (BSA). On the articular domain, when adjusted for BMI, the impact of MD on overall PsA disease activity was significant only for enthesitis. Moreover, the beneficial effects of both MD and exercise were observed independently of weight loss, suggesting that these lifestyle modifications can positively influence disease outcomes beyond mere weight management [78].

### 4.3. Ketogenic Diet

The ketogenic diet (KD) is a high-fat, low-carbohydrate diet that has gained popularity for its potential benefits in weight loss, metabolic health, and various medical conditions. This diet involves a significant reduction in carbohydrate intake and an increase in fat consumption, aiming to induce a state of ketosis in the body, which, deprived of sufficient carbohydrates, starts breaking down fats into ketones such as acetone, acetoacetate and β-hydroxybutyrate, which then serve as the primary energy source [79]. The potential beneficial effects of KD in patients with inflammatory arthritis seems to derive mainly from the reduction of systemic inflammation, through controlling factors that determine the production of proinflammatory cytokines such as excess body weight, hyperinsulinemia and activation of the NLRP3 inflammasome [80].

In this context, a recent study involving 26 obese PsA patients who followed either MD or KD for eight weeks, with a six-week washout period before switching diets for another eight weeks [81]. The authors found that both diets led to significant reductions in weight, BMI, waist circumference, total fat mass, and visceral fat, with greater results with KD. Patients on KD experienced greater improvements in disease activity scores, including reductions in joint pain and swelling, DAPSA, and PASI scores. Moreover, KD provided better control over certain inflammatory markers, such as CRP, and also improved metabolic parameters, including insulin sensitivity and lipid profiles. Conversely, MD was more effective in improving overall cardiovascular health. Therefore, while both diets can be beneficial for managing PsA and obesity, KD may offer superior anti-inflammatory benefits. However, the advantages for cardiovascular health provided by MD make it a solid alternative, particularly for long-term management.

### 4.4. Gluten-Free Diet

Gluten is a group of proteins, mainly gliadin and glutenin, that is found in wheat, barley, rice, and other grains. The amount of dietary gluten intake is not a risk factor for the development of PsA and SpA [82]. However, these patients may also have celiac disease (CD), a chronic autoimmune disorder characterized by anti-transglutaminase, anti-gliadin, and anti-endomysial autoantibodies, or Nonceliac Gluten Sensitivity (NCGS), a condition associated with a range of both gastrointestinal and non-gastrointestinal symptoms that respond to gluten restriction and recur with gluten ingestion [83]. In these cases, adhering to a gluten-free diet (GFD) can lead, in addition to controlling the intestinal disease, also to improvements in joint symptoms. In fact, especially patients with CD can frequently experience episodes of inflammatory arthritis [84].

However, recent studies highlight that the beneficial impact of a GFD on individuals without CD and NCGS is not universally applicable, and thus there is insufficient evidence to recommend a priori GFD in all SpA patients [85,86]. Further studies are needed to improve our knowledge of the association between GFD and SpA outcomes. In this context, the multicentric, randomized, placebo-controlled GlutenSpA trial (NCT04274374) is currently evaluating the impact of a 16-week GFD on the quality of life of AxSpA patients, measured with the Assessment of SpondyloArthritis International Society—Health Index (ASAS-HI) questionnaire, along with the effects on disease activity, patient-reported outcomes and gut microbiota composition [91].

Regarding the impact of other dietary interventions in SpA patients, such as a plant-based diet, the results are promising but limited. The available data largely stem from single-patient case studies, highlighting the need for more extensive research in this area [92].

### 4.5. Intermittent Fasting

Intermittent fasting (IF) is an eating pattern that alternates between periods of eating and fasting. Traditionally observed by Muslims during the month of Ramadan, IF has gained increasing attention in recent years for its potential health benefits beyond religious practice. It is now being explored as a non-pharmacological strategy for managing various chronic conditions, including musculoskeletal inflammatory disorders. The growing interest in IF stems from its potential to improve overall health, offering a complementary approach to conventional medical treatments [87]. The beneficial effects of IF on immune-mediated diseases potentially derive from several mechanisms, including a significant reduction in cytokine production, visceral fat mass, and improvements in gut microbiota composition and metabolic profile. Notably, a reduction in fasting blood glucose, triglycerides, and total cholesterol has been observed, along with an increase in the HDL/LDL ratio [88].

Regarding SpA spectrum, a multicentric study on 37 PsA patients documented a significant reduction of CRP levels, BASDAI, PASI, and DAPSA scores after one month of the IF regimen [89]. Similarly, Leeds Enthesitis Index (LEI) and dactylitis severity score (DSS) significantly decreased during the follow-up period. Moreover, another recent study evaluated the impact of IF on 56 patients with RA or SpA [90]. SpA patients experienced a significant reduction in ASDAS-ESR scores. Additionally, this study highlighted that IF does not significantly impact either adherence to or tolerance of pharmacological therapy, particularly biologics. In this context, a slight reduction in compliance was observed only with methotrexate and NSAIDs, mainly due to patients’ concerns about potential gastrointestinal side effects [90]. Despite this interesting evidence, there is still a lack of standardized recommendations on this topic, and further larger studies are needed in this field.

### 4.6. Biotic Compounds

Probiotics are specific types of foods, particularly yogurt and fermented products like kefir, sauerkraut, or kimchi, that contain beneficial microorganisms, primarily bacterial strains belonging to the *Lactobacillus* and *Streptococcus* spp. [93]. These strains must not only be present and alive at the time of consumption of the food, but they must also survive the journey through the digestive system. This includes withstanding stomach acidity and reaching the gut, where they interact with the existing microbial population. Prebiotics are non-digestible food ingredients, primarily fibers as inulin, but also conjugated linoleic acid, fructooligosaccharides, galactooligosaccharides, mannanoligosaccharide, xylooligosaccharide, and certain phenolics, that promote the growth and activity of beneficial bacteria in the gut. Unlike probiotics, which introduce live bacteria into the digestive system, prebiotics serve as food for these microorganisms, helping them thrive and successfully replicate [94]. In fact, prebiotics pass through the upper part of the gastrointestinal tract undigested and reach the colon, where they are fermented by the gut microbiota, producing short-chain fatty acids like butyrate, acetate, and propionate [95].

The constant consumption of probiotics and prebiotics is associated with various health benefits, which primarily arise from their ability to restore and maintain a healthy balance of gut microbiota. In fact, these foods help replenish beneficial bacteria that might be depleted due to different factors (e.g., poor diet, antibiotics, or illness) and inhibit the growth of other harmful bacterial species, which might compete for both nutrient supply and space to proliferate. Moreover, probiotics and prebiotics help maintain the integrity of the intestinal mucosal lining, preventing harmful substances from entering the bloodstream, and can enhance the immune system’s function by interacting with gut-associated lymphoid tissue, influencing immune responses [96].

Gut microbiota dysbiosis has been linked to the development and progression of SpA [97,98]. This action occurs through diverse complex molecular mechanisms that are still not fully understood. The main hypothesis is that this imbalance can lead to increased gut permeability, allowing bacterial antigens and toxins to enter the bloodstream, triggering the innate immune system and systemic inflammation in genetically predisposed patients, particularly those carrying the Human leukocyte antigen (HLA)-B27 haplotype [99].

Therefore, the rationale for administering these foods, especially probiotics, to SpA patients is based on the potential of these microorganisms to reduce gut inflammation by restoring the balance of gut microbiota. This restoration could, in turn, lower the systemic inflammation and mitigate SpA symptoms and course. Although the results from randomized controlled trials (RCTs) are more promising for PsO, those for SpA are not yet satisfactory [100,101]. In fact, an RCT conducted on 63 patients with active SpA demonstrated that adding orally administered probiotics—specifically containing *Streptococcus salivarius* K12, *Bifidobacterium lactis* B94, and *Lactobacillus acidophilus* L10—to conventional pharmacological therapy for 12 weeks did not result in significant improvements in physical function or joint disease activity, as measured by the BASFI and BASDAI, respectively [102]. Similarly, an oral probiotic mix containing *Lactobacillus salivarius* CUL61, *Lactobacillus paracasei* CUL08, *Bifidobacterium infantis* CUL34, and *Bifidobacterium bifidum* CUL20, administered for 12 weeks, did not produce significant improvements in global health or SpA severity compared to a placebo in an internet-based RCT involving 147 patients [103].

Postbiotics are bioactive compounds produced by probiotics, or released after their death, such as short-chain fatty acids, peptides, exopolysaccharides, and cell wall fragments, that can promote immune-modulating effects and gut barrier restoration [104]. Moreover, synbiotics are a combination of prebiotics and probiotics, designed to have a synergistic effect on the host [105]. Postbiotics and synbiotics currently lack robust data in the literature regarding their use in SpA; however, they hold potential for becoming valuable tools in the management of these patients.

### 4.7. Antioxidant Supplements

Many plant-based foods, such as berries, nuts, and various fruits and vegetables, are rich in compounds with antioxidant and anti-inflammatory properties. These elements not only help preserve the plants in their natural environment but also often exhibit antibacterial and antifungal effects [106]. Due to these properties, certain molecules from these foods are being investigated as potential adjuvants to pharmacological therapy in immune-mediated diseases, including SpA. While clinical studies are still limited, promising outcomes have been observed in murine models and experimental research.

Resveratrol, a polyphenolic compound notably abundant in red wine, reduces inflammation through several mechanisms. It inhibits pro-inflammatory enzymes, primarily cyclooxygenase-2 (COX-2), which is involved in the synthesis of prostaglandins and leukotrienes, and inducible nitric oxide synthase (iNOS). Additionally, resveratrol downregulates the production of pro-inflammatory cytokines such as TNF-α, IL-1, and IL-6. It also activates sirtuin SIRT1, which inhibits NF-κB, thereby reducing the expression of genes involved in the inflammatory response [107]. Moreover, resveratrol has strong antioxidant properties that help neutralize reactive oxygen species (ROS), which contribute to inflammation. It also reduces the activation and infiltration of immune cells, including macrophages, T cells, and neutrophils, into tissues. Additionally, resveratrol enhances autophagy, a cellular process that removes damaged cells and proteins, further contributing to its anti-inflammatory effects [108]. In murine models of AxSpA, researchers found that intragastric administration of resveratrol (20 to 50 mg/day for 4 weeks) decreased disease severity by suppressing the TLR4/NF-κB/NLRP3 pathway, restoring intestinal mucosal barrier function, and modulating gut microbiota composition [109]. Lomholt et al. investigated the anti-inflammatory effects of resveratrol, both alone and in combination with MTX or adalimumab, in synovial fluid mononuclear cells from patients with RA and SpA [110]. The study found that resveratrol, particularly when combined with MTX, significantly reduced the production of monocyte chemoattractant protein 1 (MCP-1 or CCL2), a key molecule involved in recruiting monocytes to sites of inflammation. This reduction was especially pronounced in lymphocyte-dominated cultures and in patients with low disease activity (DAS28CRP ≤ 3.2), highlighting the potential of resveratrol as an adjunct therapy in clinical settings for patients with low disease activity.

The anti-inflammatory effects of quercetin are mediated through molecular mechanisms similar to those of resveratrol. Additionally, quercetin stabilizes mast cells, preventing the release of histamine and other inflammatory mediators, and it reduces the formation of foam cells, thereby contributing to its cardioprotective effects [108]. In RA murine models, quercetin administration has been shown to improve joint inflammation. This was achieved through the inhibition of the enzyme adenosine deaminase, which helps reduce joint infiltration and neutrophil activation [111]. Additionally, quercetin promotes the apoptosis of these immune cells, contributing to its anti-inflammatory effects [112].

Curcumin, a flavonoid that is mainly found in turmeric, exerts anti-inflammatory effects through the inhibition of enzymes such as superoxide dismutase (SOD) and COX, NF-κB pathways, and enhancing the number and function of Treg [113]. In this context, an RCT demonstrated that daily administration of nanocurcumin for 4 months in AxSpA patients, through an increase in FoxP3 activation, resulted in increased Treg levels, which play a key role in maintain self-tolerance and inhibit excessive or inappropriate immune responses. Moreover, the improvement of the TReg/Th17 ratio, along with lower IL-6 levels and higher IL-10 levels detected in these patients, suggests that nanocurcumin could be an adjuvant to conventional therapies to improve the course of such condition [114]. Moreover, it was observed in vitro that the administration of curcumin in PsO and PsA patients, through the increase of serine 727 phosphorylation on STAT3, significantly attenuated the production of IL-17 by Th17 and of interferon-γ by T cells and natural killer (NK) cells [115]. Similar in vitro effects were observed with the administration of delphinidin, a type of anthocyanin found in high concentrations in foods like blueberries, grapes, and eggplants [116].

Despite their proven anti-inflammatory effects, the use of antioxidants like polyphenols and flavonoids to alleviate the symptoms of inflammatory arthritis faces several challenges in clinical practice. One major issue is their limited oral bioavailability: even when taken in high doses, only a small fraction is absorbed and utilized by the body, which diminishes their therapeutic potential. Additionally, determining the optimal dosage is complex: in vivo conditions can differ significantly from in vitro ones, and the doses required to achieve therapeutic effects in a laboratory setting may not be easily achievable through diet alone. As a result, maintaining sufficient intake often necessitates regular supplementation, which can be difficult to sustain over the long term, especially if patients do not experience immediate benefits. Furthermore, individual responses to these compounds can vary greatly due to differences in genetics, gut microbiota, and lifestyle, complicating the prediction of their effectiveness on a personal level.

## 5. Conclusions

This review highlights the important role that lifestyle factors play in the onset and progression of SpA, emphasizing that quitting smoking, moderating alcohol consumption, and adhering to a balanced diet can effectively complement conventional therapies to better control disease activity [117]. While quitting smoking can be difficult, approaches such as counseling, nicotine replacement therapies, medications, and support groups can significantly enhance success rates. Limiting alcohol intake and educating patients on potential drug interactions is recommended. Moreover, regular medical check-ups are essential for the early detection and management of both smoking- and alcohol-related issues. A balanced diet, ensuring adequate intake of calories, macronutrients, and micronutrients, is advisable, along with minimizing added sugars and ultra-processed foods. Although various dietary strategies and supplements may be promising in managing SpA, further large-scale, long-term studies are needed to confirm their sustained benefits. Overall, proactive lifestyle management, guided by healthcare professionals, is essential for improving outcomes in individuals with SpA.

## Figures and Tables

**Table 1:** Effects of smoke and alcohol on SpA onset and outcomes.

	Mechanism of Action	Effects on SpA	References
Smoking	Introduction of nicotine, carbon monoxide and free radicals that induce cellular damage and oxidative stress, leading to the release of pro-inflammatory cytokines	Increased incidence of SpA in the general population	[19,20,21,22,23,24,25,26,27,28,29,30,31,32,33,34,35,36]
		Increased disease activity and impact on global function and quality of life	
	Increase of the number and function of Th17 and decrease activity of Tregs, with increased proinflammatory activity and reduction of immunological tolerance	Greater radiographic progression	
	Negative impact on bone metabolism with reduction in BMD and increased risk of osteoporosis, fractures and structural damage	Impaired response to treatment (especially to TNFi)	
	Systemic NF-kB activation with endothelial dysfunction	Higher prevalence of comorbidities (especially cardiometabolic and osteoporosis)	
Mild-moderate alcohol intake (≤25 g/day), especially of red wine (rich in polyphenols)	Reduction in proinflammatory cytokine production	Protective effect on the development of immune-mediated and autoimmune pathologies	[37,38,39,40,41,42,43,44]
	Reduction in immune cell function and weakening of the skin-mucosal epithelial barriers	Reduction of disease activity	
	Antioxidant action with COX-2 and iNOS inhibition, ROS neutralization	Increased pain threshold	
High alcohol intake (>25 g/day)	Promotion of the activation of Th1 and Th17 cells with release of pro-inflammatory cytokines	Higher incidence of SpA and PsO in the general population	[40,41,45,46,47,48]
		Increased disease activity and impact on global function and quality of life	
	Keratinocyte proliferation	Greater radiographic progression	
	Negative impact on bone metabolism with reduction in BMD and increased risk of osteoporosis, fractures and structural damage	Impaired response to treatment (especially to TNFi)	
	Interference with the absorption of micronutrients and the metabolism of drugs	Higher prevalence of comorbidities	
		Greater risk of micronutrient deficiencies and adverse drug effects	

Abbreviations: SpA, Spondyloarthritis; Th, T Helper cells; Tregs, Regulatory T cells; BMD, Bone Mineral Density; NF-kB, Nuclear Factor kappa-light-chain-enhancer of activated B cells; TNFi, Tumor Necrosis Factor Alpha inhibitors; COX-2, Cyclooxygenase-2; iNOS, inducible Nitric Oxide Synthase; ROS, Reactive Oxygen Species; PsO, Psoriasis.

**Table 2:** Main potential advantages and disadvantages of different dietary regimes that can be adopted in SpA patients and their effects on SpA outcomes from observational studies.

Diet	Advantages	Disadvantages	Effects on SpA	Ref
Mediterranean diet	Rich in healthy fats and antioxidants	May not result in rapid weight loss compared to restrictive diets	In AxSpA and PsA patients improvement of disease activity with reduced levels of ASDAS-CRP and DAPSA	[74,75,76,77,78]
	Promotes heart health and reduces risk of cardiovascular disease			
		High-calorie foods like nuts and olive oil can lead to overeating if portions aren’t controlled		
	High in fiber, supports digestive health			
	Reduces inflammation and may lower cancer risk	Can be expensive due to reliance on fresh products	In PsA and PsO patients better control of skin symptoms with reduced PASI and BSA levels	
	Easy to follow with fewer restrictions			
Ketogenic diet	Effective for short-term weight loss and fat burning	Can cause nutrient deficiencies due to restricted food groups	In PsA patients improvement of disease activity with fewer painful and swollen joints, reduced levels of DAPSA and PASI	[79,80,81]
	May improve insulin sensitivity and blood sugar control	Keto flu (fatigue, headaches) may develop during adaptation phase		
	Helps with managing epilepsy and some neurological disorders	Long-term sustainability is difficult due to restriction on carbs	Reduction in serum CRP levels	
	May reduce hunger and increase satiety due to high-fat content	Can increase cholesterol and raise cardiovascular risk for some individuals		
	Suitable for those aiming to control blood sugar levels	Risk of digestive issues like constipation due to low fiber intake		
Gluten-free diet	Essential for individuals with celiac disease or gluten sensitivity	May lack essential nutrients (fiber, B vitamins, iron) found in whole grains	Currently limited data	[82,83,84,85,86]
		Processed gluten-free foods can be high in sugar, fat, and additives		
	Reduces digestive discomfort for those with gluten intolerance			
		Can be socially restrictive and harder to follow when eating out		
	May lead to weight loss by cutting out processed, high-calorie foods			
		Gluten-free packaged foods can be expensive		
		Unnecessary for those without gluten intolerance		
Intermittent fasting	Effective for weight loss and fat burning without calorie counting	Can lead to overeating or binge eating during feeding periods	In PsA patients improvement of disease activity with reduced levels of DAPSA, PASI, LEI, DSS, and reduced serum levels of CRP	[87,88,89,90]
	May improve lipide profile, insulin sensitivity and lower blood sugar levels	Initial side effects like fatigue, irritability, or headaches		
	Promotes autophagy	Not suitable for everyone (pregnant women, people with eating disorders)	In SpA patients ASDAS-ESR reduction	
	May reduce inflammation and support longevity	May cause nutrient deficiencies if nutrient-rich foods are not prioritized during eating windows		
	Can enhance mental clarity and focus during fasting periods	Can be difficult to maintain for those with busy schedules or active lifestyles		

Abbreviations: SpA, Spondyloarthritis; AxSpA, Axial Spondyloarthritis; PsA, Psoriatic Arthritis; ASDAS-CRP, Ankylosing Spondylitis Disease Activity Score-C-Reactive Protein; DAPSA, Disease Activity index in PSoriatic Arthritis; PsO, Psoriasis; PASI, Psoriasis Area Severity Index; BSA, Body Surface Area; CRP, C-Reactive Protein; LEI, Leeds Enthesitis Index; DSS, Dactylitis Severity Score.

## Data Availability

No new data were created or analyzed in this study.
